# Early glycoprotein IIb–IIIa inhibitors in primary angioplasty (EGYPT) cooperation: an individual patient data meta-analysis

**DOI:** 10.1136/hrt.2008.141648

**Published:** 2008-05-12

**Authors:** G De Luca, C M Gibson, F Bellandi, S Murphy, M Maioli, M Noc, U Zeymer, D Dudek, H-R Arntz, S Zorman, H M Gabriel, A Emre, D Cutlip, G Biondi-Zoccai, T Rakowski, M Gyongyosi, P Marino, K Huber, A W J van’t Hof

**Affiliations:** 1 Division of Cardiology, “Maggiore della Carità” Hospital, Eastern Piedmont University, Novara, Italy; 2 Centro di Biotecnologie per la Ricerca Medica Applicata (BRMA), Eastern Piedmont University, Novara, Italy; 3 TIMI Study Group, Cardiovascular Division, Brigham and Women’s Hospital, Boston, Massachusetts, USA; 4 Division of Cardiology, Prato Hospital, Prato, Italy; 5 Center for Intensive Internal Medicine, University Medical Center, Ljubljana, Slovenia; 6 Division of Cardiology, Herzzentrum Ludwigshafen, Ludwigshafen, Germany; 7 II Department of Cardiology, Institute of Cardiology, Jagiellonian University, Krakow, Poland; 8 Medizinische Klinik II, Kardiologie/Pulmologie, Charité, Campus Benjamin Franklin, Berlin, Germany; 9 Division of Cardiology, Hospital de Santa Maria, Lisboa, Portugal; 10 Siyami Ersek Thoracic and Cardiovascular Surgery Center, Istanbul, Turkey; 11 Interventional Cardiology Section, Beth Israel Deaconess Medical Center, Boston, Massachusetts, USA; 12 Division of Cardiology, University of Turin, Turin, Italy; 13 Department of Cardiology, Medical University of Vienna, Vienna, Austria; 14 3rd Department of Medicine (Cardiology and Emergency Medicine) Wilhelminen Hospital, Vienna, Austria; 15 Division of Cardiology, Hospital “De Weezenlanden”, Zwolle, The Netherlands

## Abstract

**Background:**

Even though time-to-treatment has been shown to be a determinant of mortality in primary angioplasty, the potential benefits from early pharmacological reperfusion by glycoprotein (Gp) IIb–IIIa inhibitors are still unclear. The aim of this meta-analysis was to combine individual data from all randomised trials conducted on facilitated primary angioplasty by the use of early Gp IIb–IIIa inhibitors.

**Methods and results:**

The literature was scanned by formal searches of electronic databases (MEDLINE, EMBASE) from January 1990 to October 2007. All randomised trials on facilitation by the early administration of Gp IIb–IIIa inhibitors in ST-segment elevation myocardial infarction (STEMI) were examined. No language restrictions were enforced. Individual patient data were obtained from 11 out of 13 trials, including 1662 patients (840 patients (50.5%) randomly assigned to early and 822 patients (49.5%) to late Gp IIb–IIIa inhibitor administration). Preprocedural Thrombolysis in Myocardial Infarction Study (TIMI) grade 3 flow was more frequent with early Gp IIb–IIIa inhibitors. Postprocedural TIMI 3 flow and myocardial blush grade 3 were higher with early Gp IIb–IIIa inhibitors but did not reach statistical significance except for abciximab, whereas the rate of complete ST-segment resolution was significantly higher with early Gp IIb–IIIa inhibitors. Mortality was not significantly different between groups, although early abciximab demonstrated improved survival compared with late administration, even after adjustment for clinical and angiographic confounding factors.

**Conclusions:**

This meta-analysis shows that pharmacological facilitation with the early administration of Gp IIb–IIIa inhibitors in patients undergoing primary angioplasty for STEMI is associated with significant benefits in terms of preprocedural epicardial recanalisation and ST-segment resolution, which translated into non-significant mortality benefits except for abciximab.

Several randomised trials^1^ have shown that primary angioplasty is superior to thrombolysis in terms of survival in the treatment of ST-segment elevation myocardial infarction (STEMI). The attempts to extend primary angioplasty to the vast majority of STEMI patients may, however, be associated with longer delays to treatment, with a negative impact on survival.^2^^–^5 Adjunctive abciximab has been shown to reduce mortality in patients undergoing primary angioplasty.^6^ ^7^ The early administration of glycoprotein (Gp) IIb–IIIa inhibitors seems even more attractive for the potential benefits expected from early recanalisation, which might overcome any potential delay to mechanical reperfusion.^8^ ^9^ The Early Glycoprotein IIb–IIIa Inhibitors in Primary Angioplasty (EGYPT) cooperation aimed at performing a comprehensive meta-analysis of randomised trials based on individual patient data to evaluate the benefits of pharmacological facilitation with Gp IIb–IIIa inhibitors in patients undergoing primary angioplasty for STEMI.

## METHODS

### Eligibility and search strategy

We identified all randomised trials comparing pharmacological facilitation by the early administration of Gp IIb–IIIa inhibitors versus its periprocedural administration in STEMI patients undergoing primary angioplasty. The literature was scanned by formal searches of electronic databases (MEDLINE, EMBASE) from January 1990 to October 2007, the scientific session abstracts in *Circulation*, *Journal of College of Cardiology*, *European Heart Journal* and *American Journal of Cardiology* from January 1990 to October 2007. The following key words were used: randomised trial, myocardial infarction, reperfusion, primary angioplasty, facilitated angioplasty, Gp IIb–IIIa inhibitors, abciximab, eptifibatide, tirofiban. No language restrictions were enforced. All principal investigators were contacted in order to provide individual patient data, which were transferred without patient identifiers (initials and birthday) to the Eastern Piedmont University, Novara, Italy. The dataset was checked for completeness and consistency and compared with the results of any publications. Queries were resolved by direct correspondence with the study investigator responsible. Data were managed according to the intention-to-treat principle.

Angiograms and ECG were not analysed by a central core laboratory, but data were provided by each principal investigator. Analysis of angiograms was based on standard definitions.^10^^–^12 In particular, distal embolisation was defined as an abrupt “cutoff” in the main vessel or one of the coronary branches of the infarct-related artery, distal to the angioplasty site.^12^ Even though ST-segment analysis was performed according to the pre-specified criteria of each trial, data were provided according to uniform thresholds (<30% no resolution; 30%–70% partial resolution; >70% complete resolution).

### Outcome measures

Angiographic endpoints were preprocedural and postprocedural Thrombolysis in Myocardial Infarction Study (TIMI) grade 3 flow distal embolisation. Myocardial perfusion was evaluated by myocardial blush grade (MBG) 3 and post-procedural electrocardiograms were evaluated for complete (>70%) ST-segment resolution. Infarct size was estimated by using peak creatine kinase levels. The primary clinical endpoint was mortality. We also analysed the rate of major bleeding complications (defined as retroperitoneal, intracranial bleeding, or a drop in haemoglobin >5 g/dl) as the major safety endpoint.

### Data analysis

Statistical analysis was performed using the Review Manager 4.27 freeware package and SPSS 15.0 statistical package. The pooled odds ratio (OR) for categorical variables was calculated by using the modified Mantel–Haenszel method with “observed minus expected” values for each trial, whereas a weighted mean difference was used for continuous variables.^24^ We performed survival analyses with the use of Cox regression analysis stratified according to trial.^25^ Survival was defined as the interval from randomisation until the event of interest. Survival curves are presented as non-stratified Kaplan–Meier across trials. Heterogeneity across trials was assessed by the I^2^ statistics. Prespecified subgroup analyses were performed according to the molecule (abciximab, tirofiban and eptifibatide). Additional subgroup analyses were performed for mortality according to diabetic status, age (>65 or <65 years) and time to treatment (>3 or <3 h), gender and infarct location (anterior versus non-anterior).

A multivariate adjustment of mortality benefits was finally performed for major clinical or angiographic characteristics, such as age, gender, diabetes, hypertension, smoking, previous revascularisation, previous myocardial infarction (MI), anterior MI, Killip class at presentation, time to treatment, time from symptom onset to Gp IIb–IIIa inhibitor administration, duration of preprocedural drug administration (from Gp IIb–IIIa inhibitor administration to balloon angioplasty), type of drug, multivessel disease, coronary stenting and interaction between molecules and early drug administration, by using a Cox regression analysis stratified according to trial (all covariates were entered in block in the model).^26^

## RESULTS

### Eligible studies

Individual patient data were obtained from 11^13^^–^17 19^–^23,27 out of 14^25^ ^28^ ^29^ trials. A total of 1662 patients were included, 840 patients (50.5%) were randomly assigned to early (administration started in the ambulance, in the community hospital before/during transportation to percutaneous coronary intervention (PCI) centres, or in the emergency room/intensive care unit of PCI hospitals) and 822 patients (49.5%) were randomly assigned to late (periprocedural) Gp IIb–IIIa inhibitor administration.

Study characteristics are reported in table 1. Baseline patient characteristics are reported in table 2. A total of six trials was conducted on abciximab (n  =  612, 36.8%), three trials on tirofiban (n  =  632, 38%) and two trials on eptifibatide (n  =  418, 25.2%). Baseline patient characteristics according to study drug are reported in table 3.

**Table 1:** Characteristics of randomised trials comparing early versus late Gp IIb–IIIa inhibitor administration in primary angioplasty

Study	Period	Study design (no of patients)	Symptom duration, hours	Stent	Primary endpoints	Follow-up duration
ReoPro-BRIDGING^13^	2003–4	Early (n = 28) versus late (n = 27) abciximab*	<6	Yes	Preprocedural TIMI 3 flow, cTFC and MACE	1 year
RELAx-AMI^14^	2003–4	Early (n = 105) versus late (n = 105) abciximab*	<6	Yes	Preprocedural TIMI 3 flow, ST resolution, myocardial salvage	30 days
Rakowski *et al*^15^	2004	Early (n = 25) versus late (n = 30) abciximab*	<12	Yes	Preprocedural TIMI 3 flow, ST resolution, LVF	1 year
ERAMI^16^	2001–2	Early (n = 36) versus late (n = 38) abciximab)*	<12	nr	Preprocedural TIMI flow	1 year
Zorman *et al*^17^	1998–2001	Early (n = 56) versus late (n = 56) abciximab*	<12	Yes	Early (60 minutes) ST-segment resolution, preprocedural 3 TIMI flow	6 months
REOMOBILE^18^	2001–2	Early (n = 52) versus late (n = 48) abciximab*	<6	Yes	Preprocedural TIMI flow	1 year
Cutlip *et al*^19^	2001–2	Early (n = 28) versus late or no (n = 30) tirofiban†	<12	Yes	Preprocedural TIMI flow	30 days
On-TIME^20^	2001–2	Early (n = 251) versus late (n = 256) tirofiban†	<6	Yes	Preprocedural TIMI flow	1 year
Emre *et al*^21^	2002–3	Early (n = 32) versus late (n = 34) tirofiban†	<6	Yes	Myocardial perfusion and functional recovery at 30 days	30 days
INTAMI^22^	2002–4	Early (n = 53) versus late or no (n = 49) eptifibatide‡	<12	Yes	Preprocedural TIMI 3 flow	1 year
TITAN-TIMI 34^23^	2004–5	Early (n = 180) versus late or no (n = 163) eptifibatide‡	<6	Yes	Preprocedural TIMI frame count	30 days

*0.25 mg/kg intravenous bolus, followed by 0.125 μg/kg per minute infusion (12 h).

†10 μg/kg intravenous bolus followed by 0.15 μg/kg per minute infusion (24 h).

‡180 μg/kg intravenous double bolus followed by 2.0 μg/kg per minute infusion (12–24 h).

cTFC, corrected TIMI frame count; Gp, glycoprotein; LVF, left ventricular function; MACE, major adverse cardiac events; nr, not reported; TIMI 3, Thrombolysis in Myocardial Infarction Study grade 3 flow.

**Table 2:** Patient demographic and clinical characteristics

Variables	Early Gp IIb–IIIa inhibitors	Late Gp IIb–IIIa inhibitors
	(n = 840)	(n = 822)
Age, years		
Median	61	61
Range	52–70	52–70
Sex, n (%)	642/840 (76.4%)	641/822 (78.0%)
Hypertension, n (%)	353/838 (42.1%)	347/822 (42.2%)
Diabetes, n (%)	123/840 (14.6%)	135/822 (16.4%)
Previous MI, n (%)	67/838 (8.0%)	80/822 (9.7%)
Previous revascularisation, n (%)	61/792 (7.7%)	59/770 (7.7%)
Smoking, n (%)	440/840 (52.4%)	419/822 (51.0%)
Hypercholesterolemia, n (%)	298/840 (35.5%)	309/820 (37.7%)
Killip class III/IV, n (%)	33/722 (4.6%)	33/705 (4.7%)
Anterior MI, n (%)	361/831 (43.4%)	369/819 (45.1%)
Symptom onset to Gp IIb–IIIa inhibitor time, minutes*		
Median	100	197
25–75th percentiles	65–178	144–275
Ischaemia time, minutes		
Median	193	203
25–75th percentiles	146–270	150–285
Infarct-related artery		
LAD, n (%)	351(41.7%)	361 (43.9%)
CX, n (%)	124 (14.7%)	100 (12.1%)
RCA, n (%)	339 (40.3%)	336 (40.9%)
GRAFT, n (%)	6 (0.7%)	6 (0.7%)
LM, n (%)	4 (0.5%)	5 (0.6%)
Multivessel disease, n (%)	437/757 (57.7%)	433/737 (58.8%)
Follow-up		
Median	330	347
25–75th percentiles	30–360	30–360

All p values are not significant except for the time from symptom onset to administration of Gp IIb–IIIa inhibitors* (p<0.001).

CX, circumflex artery; Gp, glycoprotein; LAD, left descending coronary artery; LM, left main artery; MI, myocardial infarction; RCA, right coronary artery.

**Table 3:** Patient demographic and clinical characteristics according to study drug

Variables	Early abciximab	Late abciximab	Early tirofiban	Late tirofiban	Early eptifibatide	Late eptifibatide
	(n = 302)	(n = 310)	(n = 311)	(n = 321)	(n = 227)	(n = 191)
Age, years						
Median	61	62	62	62	58	59
25–75th percentiles	52–69	52–72	54–70	52–70	50–70	51–68
Sex, n (%)	228/302 (75.5%)	239/310 (77.1%)	249/311 (80.1%)	259/321 (80.7%)	165/227 (72.7%)	143/191 (74.9%)
Hypertension, n (%)	143/301 (47.5%)	143/310 (46.1%)	97/311 (31.2%)	110/321 (34.3%)	113/226 (50.0%)	94/191 (49.2%)
Diabetes, n (%)	61/302 (20.2%)	69/310 (22.3%)	37/311 (11.9%)	42/321 (13.1%)	35/227 (15.4%)	37/191 (19.4%)
Previous MI, n (%)	14/301 (4.7%)	26/310 (8.4%)	21/310 (6.8%)	31/321 (9.7%)	32/227 (14.1%)	23/191 (12.0%)
Previous revascularisation, n (%)	9/254 (3.5%)	13/258 (5.0%)	22/311 (7.1%)	22/321 (6.9%)	30/227 (13.2%)	24/191 (12.6%)
Smoking, n (%)	151/302 (50.0%)	146/310 (47.1%)	176/311 (56.6%)	196/321 (61.1%)	113/227 (49.8%)	77/191 (40.3%)
Hypercholesterolemia, n (%)	135/302 (44.7%)	142/309 (46.0%)	78/311 (25.1%)	90/321 (28.0%)	85/227 (37.4%)	77/190 (40.5%)
Killip class III/IV, n (%)	15/302 (4.9%)	14/310 (4.4%)	3/251 (1.2%)	4/256 (1.6%)	15/169 (9.2%)	15/139 (10.8%)
Anterior MI, n (%)	153/302 (50.7%)	157/310 (50.6%)	127/311 (40.8%)	138/321 (43.0%)	81/218 (37.2%)	74/188 (39.4%)
Symptom onset to Gp IIb–IIIa inhibitor time, minutes*						
Median	130	203	64	191	150	199
25–75th percentiles	800–203	145–300	50–85	145–264	95–257	138–267
Ischaemia time, minutes						
Median	194	208	193	198	191	210
25–75th percentiles	145–271	150–300	150–249	150–270	138–295	147–303
Infarct-related artery						
LAD, n (%)	151 (50%)	153 (49.4%)	123 (39.5%)	134 (41.7%)	77 (33.9%)	74 (38.7%)
CX, n (%)	34 (11.3%)	30(9.7%)	51 (16.4%)	41 (12.8%)	39 (17.2%)	29 (15.2%)
RCA, n (%)	113 (37.4%)	122 (39.4%)	123 (39.5%)	131 (40.8%)	103 (45.3%)	83 (43.5%)
GRAFT, n (%)	0	6 (0.7%)	2 (0.6%)	4 (1.2%)	4 (1.8%)	2 (1.0%)
LM, n (%)	0	3 (1.0%)	4 (1.3%)	1 (0.3%)	0	1 (0.5%)
Multivessel disease, n (%)	120/254 (47.2%)	142/258 (55.0%)	150/282 (53.2%)	147/289 (50.9%)	167/221 (75.6%)	144/190 (75.8%)
Follow-up						
Median	180	180	357	356	30	30
25–75th percentiles	30–360	30–360	346–360	342–360	30–30	30–30

All p values are not significant except for the time from symptom onset to administration of Gp IIb–IIIa inhibitors* (p<0.001).

CX, circumflex artery; Gp, glycoprotein; LAD, left descending coronary artery; LM, left main artery; MI, myocardial infarction; RCA, right coronary artery.

### Angiographic endpoints

#### Preprocedural TIMI 3 flow

**Figure 1:**
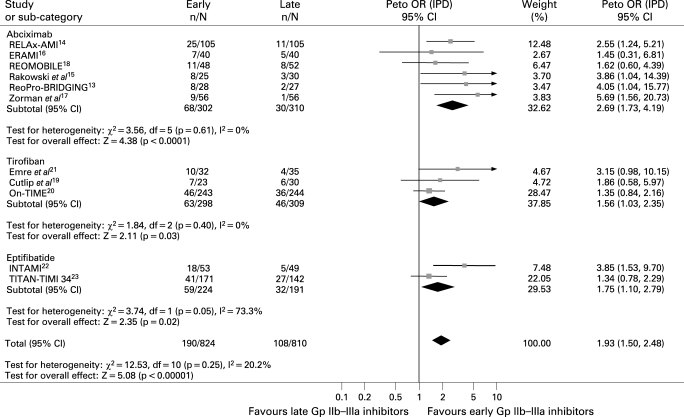
Facilitation with early glycoprotein IIb–IIIa inhibitors and preprocedural TIMI flow with Peto odds ratios and 95% CI. The size of the data markers (squares) is approximately proportional to the statistical weight of each trial. Gp, glycoprotein; IPD, individual patient data; OR, odds ratio; TIMI, Thrombolysis in Myocardial Infarction Study.

Data were available fot 1634 patients. As shown in fig 1, early Gp IIb–IIIa inhibitors were associated with a significantly improved preprocedural TIMI 3 flow (23.0% versus 13.3%, Peto OR 1.93; 95% CI 1.50 to 2.48; p<0.001, p_het_ = 0.25) with similar benefits across the three molecules.

#### Postprocedural TIMI 3 flow

**Figure 2:**
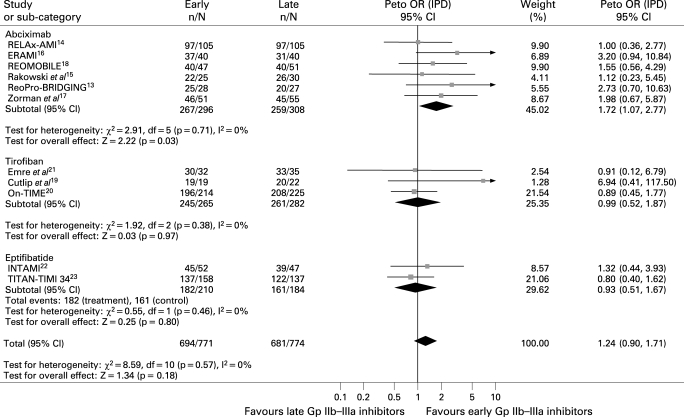
Facilitation with early Gp IIb–IIIa inhibitors and postprocedural TIMI 3 flow with Peto odds ratios and 95% CI. The size of the data markers (squares) is approximately proportional to the statistical weight of each trial. Gp, glycoprotein; IPD, individual patient data; OR, odds ratio; TIMI, Thrombolysis in Myocardial Infarction Study.

Data were available for 1551 patients. As shown in fig 2, no difference was observed in the rate of postprocedural TIMI 3 flow (90% versus 87.9%, Peto OR 1.24; 95% CI 0.90 to 1.71; p = 0.18, p_het_ = 0.57). Early abciximab was, however, associated with a significant improvement in postprocedural TIMI 3 flow (90.2% versus 84.1%, Peto OR 1.72; 95% CI 1.07 to 2.77; p = 0.03, p interaction of abciximab versus small molecules 0.057).

#### Distal embolisation

**Figure 3:**
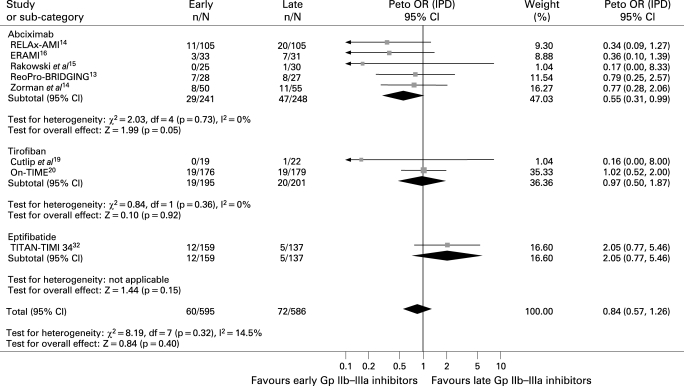
Facilitation with early Gp IIb–IIIa inhibitors and distal embolisation with Peto odds ratios and 95% CI. The size of the data markers (squares) is approximately proportional to the statistical weight of each trial. Gp, glycoprotein; IPD, individual patient data; OR, odds ratio.

Data were available for 1181 patients. Early Gp IIb–IIIa inhibitors were not associated with significant benefits in terms of distal embolisation (10.1% versus 12.3%, Peto OR 0.84; 95% CI 0.57 to 1.26; p = 0.4, p_het_ = 0.32; fig 3), except for abciximab (12% versus 19%, Peto OR 0.55; 95% CI 0.31 to 0.99; p = 0.05, p interaction of abciximab versus small molecules 0.057).

### Myocardial perfusion

#### Myocardial blush

**Figure 4:**
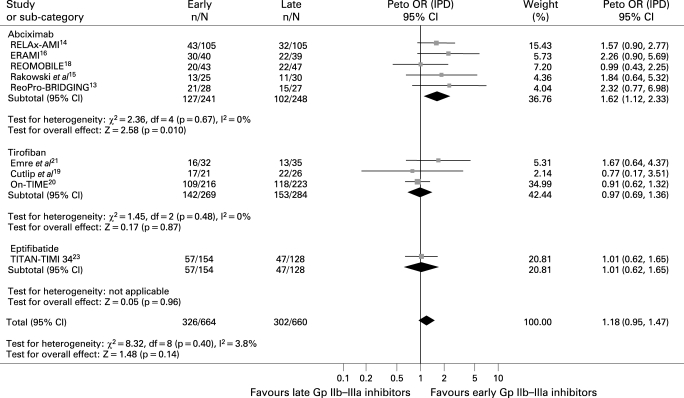
Facilitation with early Gp IIb–IIIa inhibitors and myocardial blush grade 3 with Peto odds ratios and 95% CI. The size of the data markers (squares) is approximately proportional to the statistical weight of each trial. *Myocardial perfusion evaluated by myocardial perfusion grade. Gp, glycoprotein; IPD, individual patient data; OR, odds ratio.

Data were available for 1324 patients. As shown in fig 4, early Gp IIb–IIIa inhibitors were associated with slight benefits in final MBG 3 (49.1% versus 45.8%, Peto OR 1.18; 95% CI 0.95 to 1.47; p = 0.14, p_het_ = 0.40, number needed to treat 30.3). In an analysis limited to abciximab trials, the benefits achieved statistical significance (52.7% versus 41.1%, Peto OR 1.62; 95% CI 1.12 to 2.33; p = 0.01; p interaction of abciximab versus small molecules 0.02).

#### ST-segment resolution

**Figure 5:**
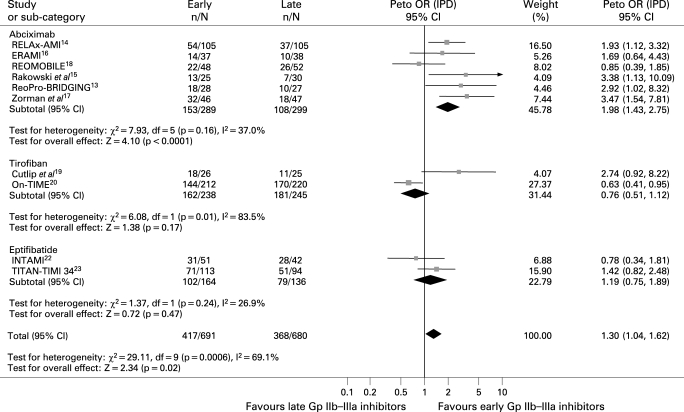
Facilitation with early Gp IIb–IIIa inhibitors and complete ST-segment resolution with Peto odds ratios and 95% CI. The size of the data markers (squares) is approximately proportional to the statistical weight of each trial. Gp, glycoprotein; IPD, individual patient data; OR, odds ratio.

Data were available for 1371 patients. As shown in fig 5, early Gp IIb–IIIa inhibitors were associated with significant benefits in terms of complete ST-segment resolution (60.3% versus 54.1%, Peto OR 1.30; 95% CI 1.04 to 1.62; p = 0.02, p_het_<0.001). This difference was greater for early versus later abciximab (52.9% versus 36.1%, Peto OR 1.98; 95% CI 1.43 to 2.75; p<0.001; p interaction of abciximab versus small molecules <0.001).

#### Enzymatic infarct size and abortion of MI

**Figure 6:**
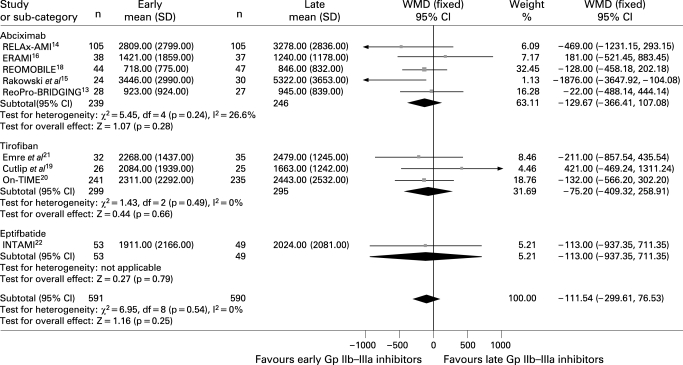
Facilitation with early Gp IIb–IIIa inhibitors and enzymatic infarct size (peak creatine kinase) with weighted mean difference and 95% CI. The size of the data markers (squares) is approximately proportional to the statistical weight of each trial. Gp, glycoprotein; OR, odds ratio; WMD, weighted mean difference.

Data on creatine kinase levels were available for 1181 patients. As shown in fig 6, early Gp IIb–IIIa inhibitors were associated with a trend in benefits in terms of enzymatic infarct size (weighted mean difference −111.5; 95% CI −229.6 to 76.5; p = 0.25, p_het_ = 0.54).

**Figure 7:**
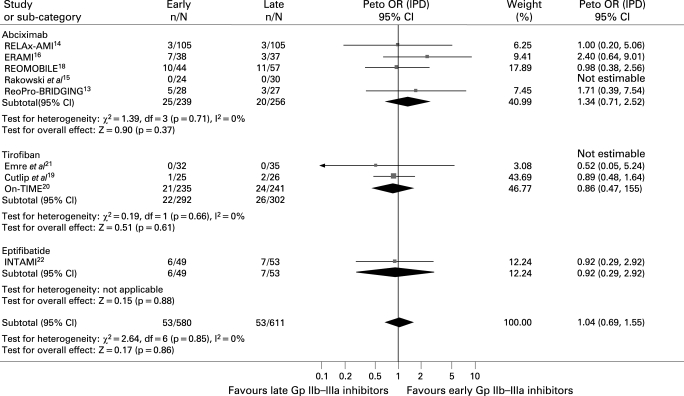
Facilitation with early Gp IIb–IIIa inhibitors and myocardial abortion with Peto odds ratios and 95% CI. The size of the data markers (squares) is approximately proportional to the statistical weight of each trial. Gp, glycoprotein; IPD, individual patient data; OR, odds ratio.

Early Gp IIb–IIIa inhibitors were associated with a non-significantly higher rate of abortion of MI (9.7% versus 8.1%, Peto OR 1.04; 95% CI 0.69 to 1.55; p = 0.86, p_het_ = 0.85; fig 7).

### Clinical endpoints

#### Mortality

**Figure 8:**
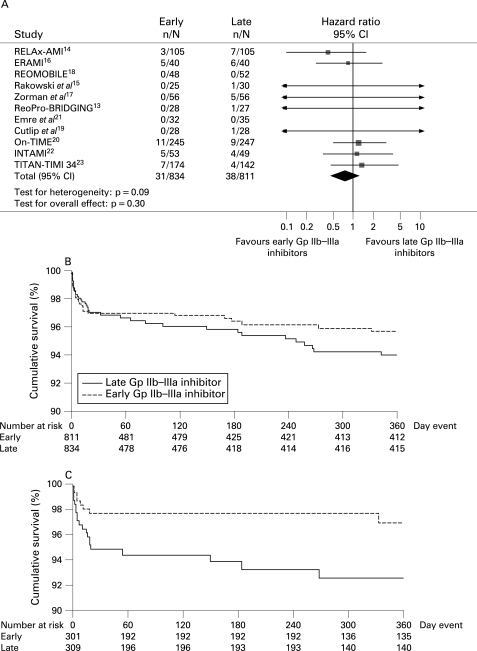
(A) Facilitation with early Gp IIb–IIIa inhibitors and mortality with pooled hazard ratios and 95% CI. The size of the data markers (squares) is approximately proportional to the statistical weight of each trial. (B) Kaplan–Meier survival curves according to early versus late Gp IIb–IIIa inhibitors. (C) Kaplan–Meier survival curves according to early versus late Gp IIb–IIIa inhibitors in trials with abciximab. Gp, glycoprotein.

**Figure 9:**
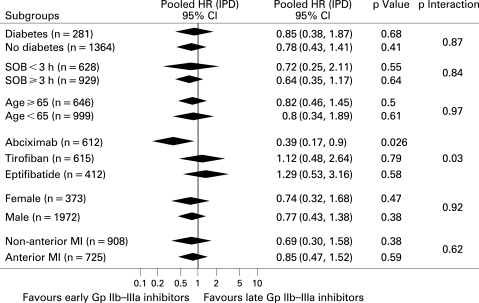
Facilitation with early Gp IIb–IIIa inhibitors and mortality with pooled hazard ratios and 95% CI in subgroups of patients. Gp, glycoprotein; HR, hazard ratio; IPD, individual patient data; SOB, symptom onset-to-balloon time.

Follow-up data were available at 30 days in four trials,^14^ ^19^ ^21^ ^23^ at 6 months in one trial^17^ and at one year in six trials^13^ ^15^ ^16^ ^18^ ^20^ ^22^ (table 1). As shown in fig 8, early Gp IIb–IIIa inhibitors were associated with non-significantly larger benefits in mortality (3.7% versus 4.7%; hazard ratio (HR) 0.78; 95% CI 0.49 to 1.26; p = 0.3, p_het_ = 0.09), which were more pronounced with abciximab (2.6% versus 6.5%; HR 0.39; 95% CI 0.17 to 0.9; p = 0.026, p_het_ = 0.76; p interaction of abciximab versus small molecules 0.034; fig 9). The results did not change for either overall or abciximab after multivariate adjustment (adjusted HR 0.32; 95% CI 0.24 to 4.13; p = 0.38; adjusted HR 0,38; 95% CI 0.15 to 1.00; p = 0.05, respectively). Additional subgroup analyses did not show any difference in treatment response according to the high-risk subsets of patients.

#### Safety endpoint

No difference was observed in terms of major bleeding complications (3.2% versus 2.9%, Peto OR 1.13; 95% CI 0.62 to 2.06; p = 0.68, p_het_ = 0.51).

## DISCUSSION

The EGYPT cooperation aimed at performing a meta-analysis to evaluate the benefits from the early administration of Gp IIb–IIIa inhibitors in patients undergoing primary angioplasty, based on individual data of 1662 patients enrolled in 11 randomised trials.^13^^–^23 The main finding of this meta-analysis is that facilitation with Gp IIb–IIIa inhibitors improved preprocedural recanalisation. In the analysis limited to the abciximab trials, there was a significant mortality reduction with early versus late abciximab administration. It is of note that other subanalyses also demonstrated improvement in postprocedural TIMI 3 flow, MBG, distal embolisation and ST-segment resolution achieved with early abciximab, although the interaction was statistically significant only for MBG and improved ST-segment resolution. Comparisons between agents must be made with extreme caution given the uncontrolled features of the trials, the possible subtherapeutic dosages utilised in some of the studies and the differences in timing of early administration. Also, it must be noted that none of the studies compared agents, so inferences are based on differences between early versus late administration for each agent. Nevertheless, this is the first demonstration of mortality benefits from pharmacological facilitation in STEMI patients undergoing primary angioplasty and indicates a need for further studies to identify the best strategy.

Recent investigations have demonstrated that time to treatment is a relevant issue in primary angioplasty, with a significant impact on mortality.^3^^–^5 It has been hypothesised that the early administration of pharmacological therapy may induce earlier reperfusion, resulting in reduced infarct size and improved survival, particularly when long-distance transportation is required.^8^ ^9^

The ASSENT-4 trial^30^ showed harmful effects from facilitation with full-dose tenecteplase in patients undergoing primary angioplasty, despite improved preprocedural recanalisation. These data have been explained by a potential intracoronary prothrombotic rebound at the time of angioplasty induced by lysis,^31^ which could be limited by the administration of Gp IIb–IIIa inhibitors. These benefits may, however, be counterbalanced by a larger incidence of bleeding complications, particularly in elderly patients.^32^ Several randomised trials have been conducted to investigate the benefits from the early administration of Gp IIb–IIIa inhibitors in patients undergoing primary angioplasty.^13^^–^23,27^–^29 As the adjunctive use of Gp IIb–IIIa inhibitors, mostly abciximab, has been shown to reduce mortality among patients undergoing primary angioplasty,^6^ ^7^ further benefits would be expected by an early reperfusion achieved by early drug administration.

A subanalysis of the Abciximab before Direct Angioplasty and Stenting in Myocardial Infarction Regarding Acute and Long-Term Follow-up (ADMIRAL) trial showed that early abciximab administration (in the emergency department or in the ambulance) did improve clinical outcome compared with late administration.^33^

In a recent meta-analysis performed on pharmacological facilitation in primary angioplasty, no overall benefits in short-term (30 days) mortality were observed with inhibitors of Gp IIb–IIIa inhibitors,^34^ and their use was discouraged by the authors in daily clinical practice, unless in randomised trials.

That meta-analysis did not include all currently available trials, however, and analysed only a restricted number of endpoints with a limited duration of follow-up. Furthermore, no prespecified subanalysis was performed according to the type of molecule. In a recent smaller meta-analysis, restricted to abciximab and including randomised trials and non-randomised subgroup analyses, early abciximab did result in significant benefits in terms of myocardial perfusion and an increased number of aborted infarctions but without significant impact on mortality compared with late administration.^35^

Although significantly improved preprocedural recanalisation was the major benefit of early Gp IIb–IIIa inhibitors, there were also suggestions of improved myocardial reperfusion. Most notably, complete ST-segment resolution was significantly better overall. Several studies have reported an association between this marker and mortality. Early abciximab, but not the small molecules, was also associated with increased MBG 3 compared with late use. The underlying mechanisms for these beneficial effects may be the diminished distal embolisation of platelet aggregates (as observed in the current meta-analysis) or inhibition of the direct interaction of platelets with the reperfused endothelium by abciximab.^36^ ^37^

The survival benefits of early Gp IIb–IIIa inhibitors did not significantly change across most of the subgroups analysed.

Disappointing results have been observed in the Facilitated Intervention with Enhanced Reperfusion Speed to Stop Events (FINESSE) trial, recently presented at the 2007 annual meeting of the European Society of Cardiology.^29^ The trial was prematurely stopped due to slow recruitment, with the inclusion of up to 2500 STEMI patients. No advantages in terms of clinical outcome were observed at 3-month follow-up with facilitation by either combotherapy (abciximab and half-dose reteplase) or abciximab, compared with late periprocedural abciximab administration, despite higher patency rates, mainly with combotherapy. It must be remarked that the FINESSE trial did include several centres with large variability in experience and skills, whereas our meta-analysis included trials mainly conducted at high-volume and highly experienced primary PCI centres. In addition, the slow recruitment rate observed in the FINESSE trial (approximately a mean of 10 patients a year enrolled per centre over 4 years) may have led to a selection bias. Finally, even though the aim of the trial was to investigate facilitation, more than 50% of patients were enrolled and randomly assigned in primary PCI centres. Longer follow-up data and a more extensive analysis will certainly provide important additional information before final conclusions can be drawn from that trial.

The Ongoing Tirofiban in Myocardial Infarction Evaluation 2 (On-TIME-2) trial,^38^ investigating the early administration of high-dose tirofiban, will certainly provide additional important data on this relevant issue.

### Limitations

We were unable to obtain individual patient data from three randomised trials,^27^^–^29 whereas the ADMIRAL trial was not included, because it did not compare early versus late administration of Gp IIb–IIIa inhibitors.

Even though the meta-analysis was based on individual patient data, this can not overcome the potential heterogeneity among trials caused by different inclusion and exclusion criteria and the fact that angiographic and ECG data were not analysed by a central core laboratory.

Enzymatic infarct size was estimated by peak creatine kinase levels, whereas the use of scintigraphic techniques would have potentially improved the results of the meta-analysis. The beneficial effects observed in terms of preprocedural recanalisation might have translated into benefits in terms of left ventricular remodelling and larger survival benefits at long-term follow-up, such as up to 3–5 years, which unfortunately were unavailable from current randomised trials.

On the basis of their prognostic implications and availability, we analysed major but not minor bleeding complications.

High-dose tirofiban has been demonstrated to provide higher inhibition of platelet aggregation, compared with a standard bolus dose, as used in trials included in the current meta-analysis and abciximab.^39^ ^40^ Whether the early administration of this therapy may provide benefits is currently tested in the ongoing On-TIME 2 trial.^38^

Several factors may have hampered the potential benefits of early eptifibatide administration, such as the restricted number of patients and trials included in the current meta-analysis, the relatively short-term follow-up (available in the vast majority of patients only at 30 days) and the shorter duration of drug administration before angioplasty, compared with other Gp IIb–IIIa inhibitors.

Moreover, it has to be pointed out that this meta-analysis was primarily performed to evaluate early versus late use of Gp IIb–IIIa inhibitors with respect to surrogate markers and clinical endpoints. Given the nature of the randomised studies included in this meta analysis, head-to-head comparisons suggesting significant benefit for early abciximab may reflect larger differences between early versus late abciximab and cannot be interpreted as reflecting the superiority of abciximab over other agents.

Finally, as the patients enrolled in the current randomised trials have for the most part been highly selected, caution should be exercised in extending the conclusion of this meta-analysis to the vast majority of STEMI patients undergoing primary angioplasty. As a result of the higher risk profile, however, at least similar benefits might potentially be expected in trial-ineligible compared with trial-eligible patients.^41^

## CONCLUSIONS

This meta-analysis shows that pharmacological facilitation with Gp IIb–IIIa inhibitors is associated with significant benefits in terms of preprocedural epicardial recanalisation. Despite these beneficial effects, however, early Gp IIb–IIIa inhibitors did translate into non-significant benefits in survival, except for abciximab, explained by the improved myocardial perfusion and less distal embolisation. Therefore, until the results of additional large randomised trials with long-term follow-up data become available, pharmacological facilitation with Gp IIb–IIIa inhibitor administration, particularly abciximab, may be considered in patients undergoing primary angioplasty for STEMI.
